# The Preparation and Evaluation of a Hydrochloride Hydrogel Patch with an Iontophoresis-Assisted Release of Terbinafine for Transdermal Delivery

**DOI:** 10.3390/gels10070456

**Published:** 2024-07-12

**Authors:** Mengfei Li, Xinghao Chen, Xiangxiang Su, Wenyan Gao

**Affiliations:** School of Pharmacy, Hangzhou Medical College, Hangzhou 310013, China; lmfdyx3319@126.com (M.L.);

**Keywords:** terbinafine hydrochloride, hydrogel patch, iontophoresis, transdermal delivery

## Abstract

**Background:** Terbinafine hydrochloride (TEB) is a broad-spectrum antifungal medication commonly used to treat fungal infections of the skin. This study designed a hydrogel patch assisted by an iontophoresis system to enhance the transdermal permeability of TEB, enabling deeper penetration into the skin layers. **Methods:** The influences of current intensity, pH levels, and drug concentration on the TEB hydrogel patch’s permeability were explored using an adaptive ion electroosmosis system. The pharmacokinetic profile, facilitated by iontophoresis for transdermal permeation, was analyzed through the application of microdialysis technology. Scanning electron microscopy and transmission electron microscopy were employed to assess the impact of ion electroosmotic systems on skin integrity. **Results:** The cumulative drug accumulation within 8 h of the TEB hydrogel patches, assisted by iontophoresis, was 2.9 and 7.9 times higher than without iontophoresis assistance and TEB cream in the control group, respectively. TEB hydrogel patches assisted by iontophoresis can significantly increase the permeability of TEB, and the AUC_(0–8 h)_ was 3.4 and 5.4 times higher, while the C_max_ was 4.2 and 7.3 times higher than the TEB hydrogel patches without iontophoresis, respectively. This system has no significant impact on deep-layer cells. **Conclusions:** This system may offer a safe and effective clinical strategy for the local treatment of deep antifungal infections.

## 1. Introduction

**Figure 1:**
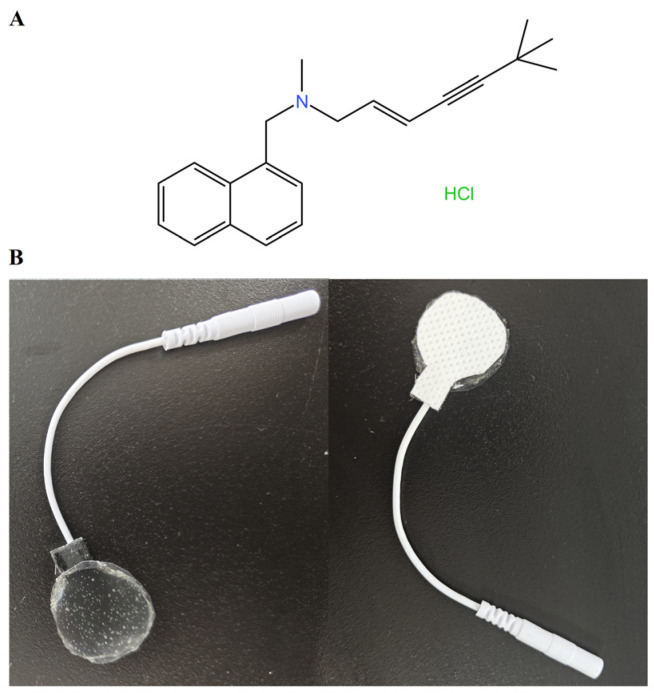
Chemical structure of terbinafine hydrochloride (**A**) and terbinafine hydrochloride hydrogel patch (**B**).

Terbinafine hydrochloride (TEB) (Figure 1A) is an allylamine antifungal agent used for the treatment of various infectious diseases, such as onychomycosis, tinea corporis, and tinea cruris, through inhibiting squalene epoxidase and interfering with ergosterol synthesis [1,2]. However, with continuous research in clinical pharmacy, the oral administration of terbinafine has induced many adverse reactions, including drug interactions, hepatotoxicity, gastrointestinal, and systemic side effects [3,4,5]. Fortunately, local administration can improve patient compliance, such as targeted therapy, sustained release, and prolonged drug action time. Moreover, it can overcome various limitations and side effects associated with oral administration. Therefore, the local administration of terbinafine has become a primary focus in pharmaceutical formulation development [6,7,8]. However, its inadequate transdermal permeability poses challenges in achieving effective therapeutic concentrations at the site of local treatment. Addressing this issue requires further research and intervention to enhance the efficacy of TEB in local antifungal therapy [9,10,11]. Moreover, more research needs to be explored to understand how to improve the local delivery mechanism of TEB, enhance its permeability, and ensure that the drug is continuously released at the treatment site [12,13,14]. These efforts may help to overcome the limitations of TEB treatment and thereby improve the effectiveness of antifungal therapy.

Terbinafine is currently available in free base and hydrochloride formulations [15], as well as topical preparations, including creams, gels, and sprays. TEB is commonly administered as terbinafine hydrochloride in clinics, with the molecular formula C_21_H_26_ClN and the molecular weight (MW) of 327.9 g/mol. Its melting point is 204–208 °C, and it has an oil–water partition coefficient (LogP) of 3.3. TEB•HCl is a white powder, easily soluble in solvents like methanol, dichloromethane, and ethanol, and has a slightly lower solubility in water [16]. However, the limited local concentration of terbinafine poses a significant obstacle in effectively treating deep-skin fungal infections [15]. In order to address this challenge, researchers have proposed various strategies. One approach involves the use of liposomes as carriers to enhance skin permeability, encapsulation efficiency, and drug stability [17]. Another promising avenue of exploration is the utilization of nanovesicles as potential carriers [18]. However, these approaches often entail complex preparation processes, have limited stability, and rely on passive drug transport, leading to variable therapeutic effects among individuals.

Due to the barrier function of the stratum corneum, skin administration is often limited, which impedes the passage of most drug molecules, allowing only a few with specific physicochemical properties to penetrate the skin [19]. Iontophoresis is a technique that enhances the transdermal delivery of compounds by applying a safe and small electric current, primarily used for the delivery of large and charged molecules [20,21]. It is an active non-invasive drug delivery technology that facilitates the transport of charged and neutral molecules into and across biological membranes [22,23]. The preparation of drugs into suitable formulations and their compatibility with iontophoresis can enhance the transdermal permeation and therapeutic efficacy of the drugs [24,25]. By adjusting the pH, terbinafine can be transformed into an ionic drug. Currently, there are no relevant products or reports on TEB hydrogel patches that are assisted by iontophoresis systems.

This study aims to develop terbinafine into hydrogel patches (Figure 1B) and adapt terbinafine hydrogel patches to an iontophoresis device to increase the skin drug concentration of terbinafine. Hydrogel patches have the advantages of high drug loading, precise dosing, and broad applicability, and they exhibit good compatibility with human skin. The back of the hydrogel patch is covered with a conductive backing layer, which prevents reductions in the effective drug dosage due to friction from clothing or other external factors. It is expected that this approach will significantly improve the local therapeutic concentration of the drug.

## 2. Results and Discussion

### 2.1. Establishment and Verification of TEB HPLC Methodology

**Figure 2:**
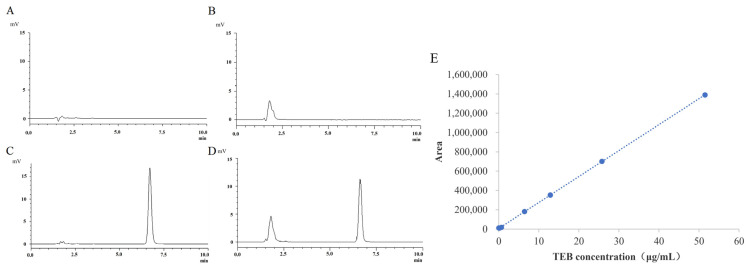
HPLC chromatogram for specificity investigation. (**A**) Blank mobile phase (methanol–water (0.2% triethylamine and 1% acetic acid) = 7:3). (**B**) 20% PEG 400-20 mM NaH_2_PO_4_. (**C**) Reference substance of TEB (6.25 μg/mL, Dissolve in NS). (**D**) Reference substance of TEB (5.85 μg/mL, Dissolve in 20% PEG 400-20 mM NaH_2_PO_4_). (**E**) Standard curve of TEB transdermal receiver solution.

Specificity: The chromatograms of the blank mobile phase solution, blank 20% PEG400-20 mM NaH_2_PO_4_ transdermal receiving solution, and TEB reference solution (prepared with NS and 20% PEG400-20 mM NaH_2_PO_4_ solution) are shown in Figure 2A–D. The chromatograms confirmed that the mobile phase solvent and the transdermal receiving solution did not interfere with terbinafine detection under the specified chromatographic conditions [26]. This indicated that the method employed was highly specific and fulfilled the requirements for sample detection.

Linear range: A TEB reference solution was diluted step by step into a control solution with concentrations of 50, 25, 12.5, 6.25, 0.625, 0.125, and 0.025 μg·mL^−1^, respectively, and the peak area was recorded using HPLC. The peak area (A) was used for the linear regression of TEB concentration (C), and the regression equation, correlation coefficient, and linear graph were recorded. As shown in Figure 2E, regression equation A = 26864C + 6874.7 and regression coefficient R_2_ = 1.0, indicating a good linear relationship between TEB concentration and peak area in this concentration range.

Precision and accuracy: Intra-day and inter-day precision were evaluated using reference solutions with TEB concentrations of 25.0, 12.5, and 6.25 μg·mL^−1^. The results demonstrated excellent precision and accuracy, as evidenced by the relative standard deviation (RSD) values below 2% (Table 1). These findings met the requirements for sample analysis.

Detection limit and quantitation limit: After processing and analyzing the HPLC data, it was found that when the TEB concentration was approximately 0.02635 μg·mL^−1^, the S/N ratio reached 3. Similarly, when the concentration was around 0.13175 μg·mL^−1^, the S/N ratio was approximately 10. Based on these results, the detection limit for TEB was set at 0.02635 μg·mL^−1^, while the quantitation limit was determined to be 0.13175 μg·mL^−1^.

### 2.2. Solubility of TEB in Different Media

The solubility results are presented in Table 2. TEB exhibited a solubility of 6.32 mg·mL^−1^ in water and 5.05 mg·mL^−1^ in glycerol, indicating good solubility and facilitating the preparation of the hydrogel patches. While the solubility in the normal saline (NS) solution was lower than that in water, it was still sufficient to maintain the leaky state under physiological conditions. Moreover, it was noteworthy that the solubility of TEB was closely linked to pH, with higher pH values resulting in increased solubility.

### 2.3. Preparation and Optimization of TEB Hydrogel Patches

A three-factor and two-level factor design was employed to optimize the formulation of the TEB hydrogel patches, with initial adhesion, moisture retention, and body sensation scores used as evaluation criteria. As shown in Table 3, the results revealed that Prescription ② achieved the highest overall score. This prescription consisted of 0.625 g PVP K90, 2.0 g sodium polyacrylate/aluminum glycolate, and 0.5 g gelatin. It was important to note that when the sodium polyacrylate/aluminum glyoxyl content was too high (as in Prescription ⑧), the adhesive consistency became excessive, making it difficult to prepare the patches [27].

### 2.4. The Effect of Different Factors Influencing the Iontophoresis-Assisted Transdermal Permeation of TEB In Vitro

**Figure 3:**
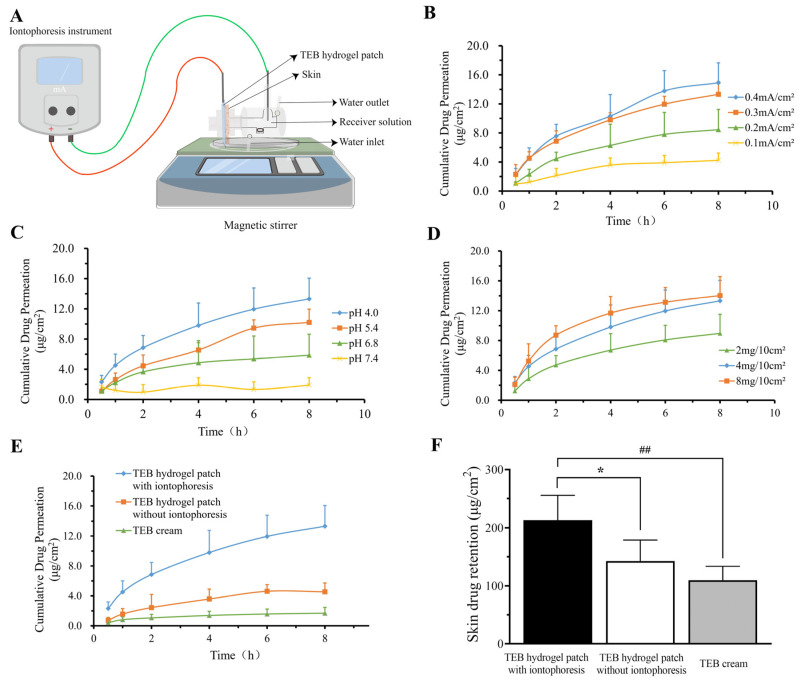
The effect of different factors influencing the iontophoresis-assisted transdermal permeation of TEB in vitro. (**A**) Schematic diagram of iontophoresis transdermal delivery in vitro. (**B**) The effect of current density on the iontophoresis penetration of TEB hydrogel patches (n = 4). (**C**) The effect of pH on the iontophoresis penetration of TEB hydrogel patches (n = 4). (**D**) The effect of drug concentration on the iontophoresis penetration of TEB hydrogel patches (n = 4). (**E**) A comparison of different TEB formulations of percutaneous penetration (n = 4). (**F**) The skin retention of different TEB formulations. * *p* < 0.05, as compared with the group of TEB hydrogel patches without iontophoresis; ^##^ *p* < 0.01, as compared with the TEB cream group.

In the in vitro skin penetration experiment (Figure 3A), cumulative permeability (Q) and time (t) were fitted using a logarithmic function, and the permeability curves demonstrated good consistency, with regression coefficients (R^2^) close to 1 [28]. To investigate the effect of different current densities on the transdermal penetration of TEB, the iontophoresis system current density was adjusted to 0.1, 0.2, 0.3, and 0.4 mA·cm^−2^. The results showed that as the current density increased from 0.1 to 0.3 mA·cm^−2^, both the cumulative permeability per unit area (Q_8h_) and the penetration rate (J_ss_) in 8 h demonstrated a gradual increase (Figure 3B and Table 4). However, when the current density increased from 0.3 to 0.4 mA·cm^−2^, the increase in Q_8h_ was not significant. Moreover, the higher current density had a damaging effect on the skin. Therefore, the optimal current density was determined to be 0.3 mA·cm^−2^.

TEB hydrogel patches with substrate pH values of 4.0, 5.4, 6.8, and 7.4 were prepared and subjected to ionic electroosmosis at a current density of 0.3 mA·cm^−2^. The results demonstrated that TEB had better a transdermal permeability at lower substrate pH values (Figure 3C). However, TEB could hardly penetrate the skin at higher pH levels. This could be due to the fact that TEB existed as an ionized form under acidic conditions, allowing it to pass through the skin via electroosmosis. Under alkaline conditions, TEB molecules were predominantly in a non-ionized form, resulting in weaker electrode attraction. Since the minimum pH that human skin can tolerate is approximately 4.0, the pH of the TEB hydrogel patch matrix was set to 4.0 [29,30].

Additionally, three TEB hydrogel patches were prepared with drug concentrations of 2, 4, and 8 mg·10 cm^−2^, respectively. When the drug content was 4 mg·10 cm^−2^, the transdermal state of the drug reached saturation (Figure 3D). Therefore, the optimal parameters for the TEB hydrogel patch ion electroosmotic system were determined as follows: current density of 0.3 mA·cm^−2^, substrate pH of 4.0, and drug concentration of 4 mg·10cm^−2^. The TEB hydrogel patch system with iontophoresis was compared with a commercial cream under optimal conditions using a skin penetration test. The results (Figure 3E and Table 4) showed low Q_8h_ and J_ss_ values for the TEB cream and TEB hydrogel patches without current loading. However, when a current was applied to the TEB hydrogel patches (0.3 mA·cm^−2^), the permeability of TEB significantly increased. The Q_8h_ and J_ss_ values were measured as 13.32 ± 4.74 μg·cm^−2^ and 3.98 μg·cm^−2^·h^−1^, respectively. The antifungal efficacy of TEB depends on the drug concentration in the affected skin site, and specifically the amount of drug retained in the skin [31,32,33]. The higher the drug retention, the greater the efficacy. The drug skin retention in the iontophoresis group was significantly higher than that in the commercially available cream and non-iontophoresis group (Figure 3F) at 8 h after administration. This indicated that the iontophoresis system can significantly promote the entry of TEB into skin tissue.

### 2.5. Percutaneous Pharmacokinetics with Microdialysis in Rat Skin

**Figure 4:**
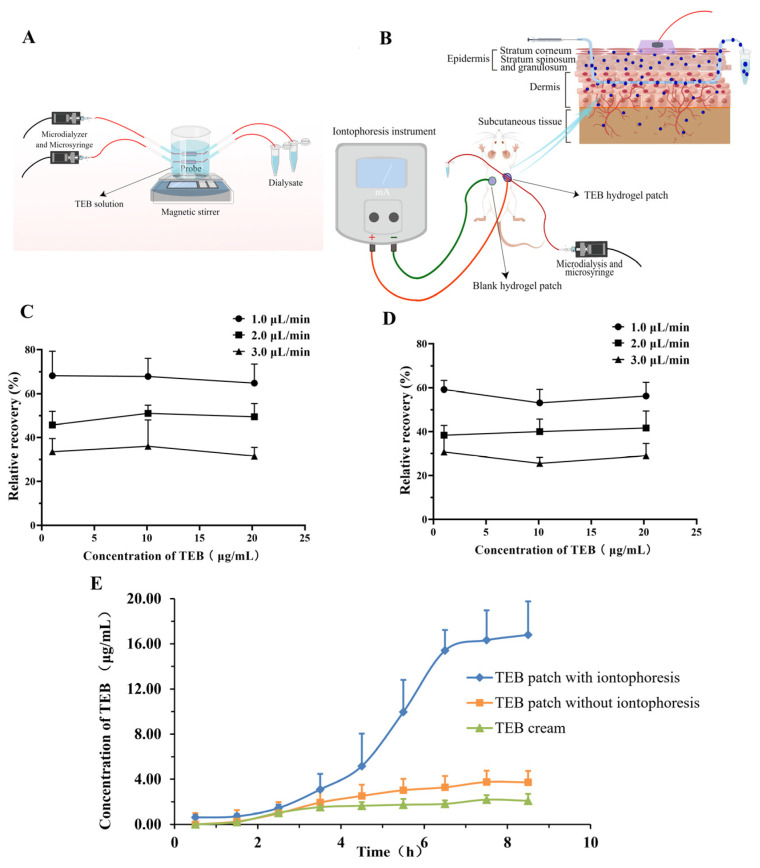
Microdialysis recovery rate of TEB in vitro and in vivo, and percutaneous pharmacokinetics. (**A**) Schematic diagram of microdialysis recovery rate in vitro. (**B**) Schematic diagram of iontophoresis transdermal delivery and microdialysis in vitro. (**C**) The effect of different flow rates and TEB concentrations on the recoveries of probes in vitro (retrodialysis, n = 4). (**D**) The effect of different flow rates and TEB concentrations on the recoveries of probes in vivo (retrodialysis, n = 4). (**E**) Percutaneous pharmacokinetics of different TEB formulations (n = 6).

Microdialysis, a novel biological sampling technique, offers various advantages, including continuous sampling, dynamic observation, quantitative analysis, a small sample size, and minimal tissue damage [34,35]. In the in vitro microdialysis recovery experiment (Figure 4A,B), the results indicated no significant difference in probe recovery among three TEB solutions with different concentrations (1.0, 10.0, and 20.0 μg·mL^−1^) at the same flow rate (*p* > 0.05) (Figure 4C). However, the flow rate had a significant impact on the recovery rate of the probe. As the flow rate increased from 1.0 to 3.0 μL·min^−1^, the recovery rate decreased from 66.9% to 36.2%, possibly due to a more efficient material exchange between the probe and the solution at lower flow rates. Similarly, the recovery rate of TEB microdialysis was closely related to the flow rate, and the recovery rate remained almost unchanged within the concentration range of 1 to 20.0 μg·mL^−1^ (Figure 4D). Therefore, a flow rate of 1.0–3.0 μL·min^−1^ should be chosen to maximize the recovery rate of TEB while still meeting detection requirements.

According to the subcutaneous pharmacokinetic data in SD rats (Figure 4E, Table 5), the TEB hydrogel patches with iontophoresis significantly enhanced the transdermal absorption of TEB. Their AUC_0–8 h_ (area under the concentration–time curve from 0 to 8 h) and C_max_ (maximum concentration) were much higher than those of the TEB patches without iontophoresis and of the TEB cream group. The AUC_0–8 h_ was 3.4 and 5.4 times higher, and the C_max_ was 4.2 and 7.3 times higher, respectively (Table 5). Additionally, the TEB hydrogel patches with iontophoresis could maintain a high subcutaneous drug concentration even after 8 h of administration, demonstrating its sustained-release properties.

### 2.6. Effect of Iontophoresis on Skin Microstructure

**Figure 5:**
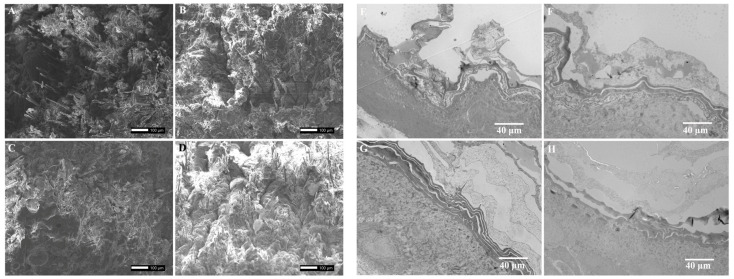
The effect of iontophoresis on the skin microstructure. (**A**–**D**) The ultrastructural damage of the epidermis under a scanning electron microscope (SEM) ((**A**) skin of normal rats (control); (**B**) the skin of rats with a TEB hydrogel patch; (**C**) skin of rats with a TEB hydrogel patch under iontophoresis; (**D**) skin of rats with the TEB cream); (**E**–**H**) deeper damage to skin cells under a transmission electronic microscope (TEM) ((**E**) skin of normal rats (control); (**F**) skin of rats with a TEB hydrogel patch; (**G**) skin of rats with a TEB hydrogel patch under iontophoresis; (**H**) skin of rats with the TEB cream).

The stimulation effect of the TEB hydrogel patches with the iontophoresis system on skin epidermal cells was observed using the SEM. The results revealed that after 8 h of administration, residual hair, textured folds, and dander on the rat skin surface were clearly visible under the SEM (Figure 5A–D). The skin treated with iontophoresis exhibited a hydration effect (Figure 5C), which could be the reason for the enhanced transdermal drug absorption by iontophoresis [21,36,37]. All skin structures appeared intact without surface cell damage or tissue swelling, indicating the good skin safety of the hydrogel patch assisted with iontophoresis [38]. There was no significant cuticle exfoliation on the skin in the TEB hydrogel patches group, the TEB hydrogel patches with iontophoresis group, or the cream group (Figure 5E–H). Additionally, no significant expansion of the space between the cuticle and the dermis was observed.

## 3. Conclusions

In this study, a TEB hydrogel patch was designed and optimized based on its properties and moisturizing capabilities. The quality of the hydrogel patches was assessed through appearance, drug content, adhesion, and other parameters. Furthermore, an iontophoresis system was incorporated to enhance the skin penetration of the drug. Compared to commercially available TEB creams and gels, the hydrogel patches offered advantages such as prolonged drug administration, increased skin affinity, and higher moisture content. The iontophoresis system further improved the drug’s retention in the skin, which was confirmed by microdialysis in vivo. These results provided valuable experimental data and a theoretical foundation for future research on this type of drug. Further investigations will explore the pharmacodynamics of the hydrogel patches to provide more extensive data for clinical research. It is anticipated that this study will enhance the local efficacy of the drug and provide new insights for the development of novel dosage forms for treating deep-skin fungal infections.

## 4. Materials and Methods

### 4.1. Rats and Reagents

Female SD rats, 200 ± 50 g, were sourced from the Laboratory Animal Center of Zhejiang Province (Hangzhou, China). They were accompanied by a production license with the number SCXK (Zhejiang 2019-0002), as well as a use license with the number SYXK (Zhejiang 2019-0011).

Terbinafine hydrochloride (purity: 99.0%, Macklin (Shanghai, China), #C12641041), Methanol (Chengdu Kelong (Chengdu, China), #2021121301), glacial acetic acid (Chengdu Kelong, #2021031601), Triethylamine (Aladdin, China, #11309090), sodium polyacrylate (Showa Denko, #161870A, Tokyo, Japan), gelatin (Rousselot (Ghent, Belgium), #1456799), Povidone K90 (IPS, America, #03600162502), glycerin (Macklin, China, #C10087952), aluminum glyoxyl (Macklin, China, #C12871941), and EDTA-2Na (Chengdu Kelong, China, #2021032201) were also obtained.

### 4.2. Establishment and Verification of TEB HPLC Analysis Method

Following the Chinese pharmacopeia (2020) and the characteristics of transdermal drug delivery preparations, this study improved the high-performance liquid chromatography (HPLC) method for TEB detection. The chromatographic conditions were as follows. Column: Diamonsil-C18 (4.6 mm × 250 mm, 5 μm), injection volume: 20 μL, column temperature: 30 °C, mobile phase: methanol–water (0.2% triethylamine and 1% acetic acid) = 7:3, detection wavelength: 282 nm, and detection time: 10 min. To ensure the scientific validity and accuracy of the experimental research, the specificity, linearity, precision, accuracy, detection limit, and quantitation limit of this method were validated.

### 4.3. Solubility Measurement of TEB in Different Media

The solubility of TEB in different solution media was determined using the saturated solution method. Excess TEB was added to various media and shaken overnight at 25 °C, and then the saturated solution was filtered, diluted, and subsequently injected into HPLC for detection.

### 4.4. Preparation of Hydrogel Patch

**Figure 6:**
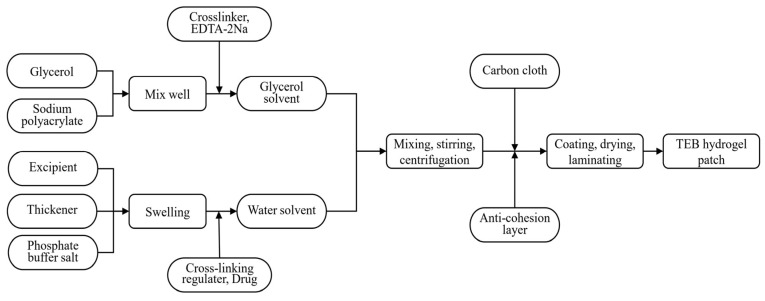
The preparation process of a TEB hydrogel patch.

The TEB gel patches were prepared using a two-phase mixing method. In total, 10 g of glycerol was weighed into beakers, and the corresponding amounts of sodium polyacrylate, aluminum glycerolate, and 0.0125 g EDTA-2Na were slowly added and stirred to obtain the glycerol phase. In another beaker, 50 mL phosphate-buffered solution (20 mM) was combined with the prescribed amounts of gelatin and PVP K90, which was heated and stirred to achieve complete swelling. After cooling, 0.125 g tartaric acid was added, followed by 0.1 g TEB, which was stirred evenly into the matrix. The glycerol phase was then slowly added to the water phase and stirred at a speed of 300 r·min^−1^. The mixture was centrifuged at 5000 r·min^−1^ for 15 min to remove any bubbles, coated onto a non-woven backing, and dried at 50 °C. The final product, a hydrogel patch, was obtained by applying an anti-cohesion layer to the coated surface (Figure 6).

### 4.5. Optimization of TEB Hydrogel Patch

In this study, we conducted a preliminary screening and identified sodium polyacrylate/aluminum hydroxide as the skeleton material for the hydrogel patch, glycerin as the humectant, gelatin as the excipient, PVP K90 as the adhesive, and the phosphate buffer as the solvent for pH adjustment and for increasing the conductivity of the gel patch. Based on previous experiments [39], it was determined that the contents of sodium polyacrylate/aluminum hydroxide, gelatin, and PVP K90 had the greatest impact on the hydrogel patch. To screen and optimize the prescription, a factorial design method was employed, utilizing a 3-factor 2-level test (Table 6).

Furthermore, we designed an evaluation index for the TEB hydrogel patches by referring to the relevant literature on hydrogel patches and considering the characteristics of the prepared TEB hydrogel patches. The evaluation index is presented in Table 7 and served as the basis for assessing the prescription of the TEB hydrogel patches. The method used to evaluate initial adhesion was carried out according to the Chinese pharmacopeia (2020). This method determined the maximum number of standard steel balls that the patch could adhere to. The moisturizing properties of the hydrogel patch were assessed by measuring its ability to retain moisture at a certain temperature, known as moisture retention. The specific experimental method involved placing the hydrogel patch in an oven at 40 °C for 24 h, weighing it before and after, and calculating the weight change. The calculation formula for moisture retention was as follows:

Moisture retention = (1 − (initial weight − final weight)/initial weight) × 100%

The initial adhesion and moisture retention scores were combined with a quantitative weight standard score for somatosensory evaluation to calculate a comprehensive score for the patch.

### 4.6. Iontophoresis-Assisted Transdermal Permeation In Vitro

The SD rats were anesthetized and euthanized, and their backs were prepared by removing the hair. The skin was then carefully excised and placed on a glass plate. Then, the subcutaneous tissue and adhesions were meticulously removed. The skin was cut into 1.5 cm × 1.5 cm squares. Next, the skin squares were positioned at the opening of the diffusion cell, with the epidermis facing outward. TEB patches were applied to the rat skin’s epidermis. The hydrogel from the ion electroosmotic group was transferred to a carbon cloth electrode, which was connected to the positive electrode of the electroosmometer. The diffusion cell cover was secured using a clamp. In the receiving cell, 4 mL of a 20% PEG400-NaH_2_PO_4_ solution was added and connected to the negative electrode of the electroosmometer. The diffusion cell was maintained at a constant temperature of 32 °C, with the stirring speed set at 600 r·min^−1^. At designated time points (0.5, 1, 2, 4, 6, and 8 h), 1 mL samples was collected, and an equal volume of blank acceptor was added after each collection. The samples were subsequently filtered using a 0.22 μm microporous filter membrane and then injected into the HPLC for detection.

The calculation formula is as follows:

$$Q_{n}=\left(C_{n}\times 4\;\mathrm{mL}+\overset{n-1}{\underset{n=1}{\sum }}n-1\times 1\;\mathrm{mL}/0.64\;{\mathrm{cm}}^{2}\right)$$

where “*n*“ represented the specific sampling time point in a series of sequential sampling intervals, and 0.64 cm^2^ was the area of the diffusion cell mouth.

Subsequently, the transdermal penetration of TEB was evaluated by conducting skin penetration experiments on isolated rats to investigate the effects of current density, hydrogel patch substrate pH, and drug concentration. The transdermal penetration performance of TEB was compared with that of a commercial TEB cream as a reference.

### 4.7. Microdialysis and Subcutaneous Tissue Pharmacokinetics In Vivo

In vitro recovery rate: Different concentrations (1.0, 10.0, and 20.0 μg·mL^−1^) of TEB saline solution were placed in a double-channel beaker. A linear probe with specific dimensions (φ = 15 μm, L = 200 μm, and 20 KDa) was immersed in the TEB saline solution through one channel of the beaker, while magnetic stirrers were placed in the beaker to ensure thorough mixing. The solution in the beaker was completely exchanged with the linear probe. Subsequently, blank normal saline was injected at different flow rates (1.0, 2.0, and 3.0 μL·min^−1^) using a microsyringe. After allowing for a 0.5 h equilibrium period, dialysate samples were collected. Four samples were collected for each flow rate. The recovery rate of TEB microdialysis was determined by calculating the ratio between the concentration of the receiving solution and the concentration of the drug in the beaker.

In vivo recovery rate: In vivo microdialysis experiments employed the reverse method, as it was not possible to directly measure the drug concentration outside the probe in the unique in vivo environment. The SD rats were anesthetized using an intraperitoneal injection of urethane (1.25 g∙kg^−1^). After removing the hair at the administration site on the abdomen, the rats were placed on a thermostatic pad at 37 °C. The linear probe was implanted into the deep dermis using a guide needle, with the probe membrane remaining in the subcutaneous tissue. Terazosin hydrochloride normal saline solutions with different concentrations (1.0, 5.0, and 10.0 μg·mL^−1^) were perfused at different flow rates (1.0, 2.0, and 3.0 μL·min^−1^). Four samples were collected at each flow rate, and a 0.5 h balance period was maintained before each collection. The recovery rate was determined by calculating the ratio between the concentration of the dialysate and the concentration of the original drug.

Transcutaneous pharmacokinetic study: The microdialysis probe membrane remained in the subcutaneous tissue of the rat, while a self-made hydrogel patch (containing 1 mg·cm^−2^, with an administration area of 2 cm × 2 cm) was applied to the hairless abdomen of the rat, directly above the microdialysis probe. The blank normal saline solution was perfused at a flow rate of 1.0 μL·min^−1^. After allowing for a 0.5 h equilibrium period, one acceptor solution was collected every hour, resulting in eight samples collected at each flow rate over a total of eight hours. The subcutaneous TEB concentration correction was determined by calculating the ratio between the dialysate concentration and the recovery rate at the corresponding time points in vivo.

The corrected concentration data were analyzed using DAS 2.0 to derive relevant parameters and generate the subcutaneous drug concentration–time curve.

### 4.8. Effects on Skin Microstructure

Transmission electron microscopy (TEM) was employed to examine the ultrastructure influences and pathological changes in the longitudinal subcutaneous tissue. The skin from the rats was removed, fixed overnight in a 2.5% glutaraldehyde solution at a temperature of 4 °C, and then processed. After cleaning, the samples were treated with osmic acid for 1 h, followed by ethanol and isoamyl acetate treatment, drying, coating, and observation under the scanning electron microscope.

### 4.9. Statistical Analysis

The statistical analysis was performed by using GraphPad Software 9.0. The data were expressed as means ± standard deviations (SDs). The statistically significant differences were determined by one-way analysis of variance (ANOVA) testing. Significance was defined as *p* < 0.05.

## Figures and Tables

**Table 1:** Precision and accuracy.

Concentration Spiked (μg/mL)	Concentration Measured (μg/mL)	Accuracy/%	RSD/%
Intra-day (n = 6)			
6.25	6.150 ± 0.020	100.582 ± 0.773	0.773
12.50	12.894 ± 0.200	103.150 ± 1.548	1.548
25.00	25.149 ± 0.020	98.397 ± 1.002	1.002
Inter-assay (n = 6)			
6.25	6.204 ± 0.119	99.270 ± 1.896	1.910
12.50	3.067 ± 0.067	103.873 ± 1.380	1.328
25.00	78.910 ± 0.415	100.848 ± 0.735	0.728

**Table 2:** The solubility of TEB in different solvents (25 °C).

Solvent	Solubility (mg/mL)	Solvent	Solubility (mg/mL)
H_2_O	6.32	NS	1.56
1,2-PG	29.82	Glycerol	5.05
PBS (pH 4.07)	6.13	20%PEG400-PBS (pH 4.07)	21.72
PBS (pH 7.11)	4.32	20%PEG400-PBS (pH 7.11)	10.96

**Table 3:** Prescription dosage screening results (points).

Prescription	Initial Adhesion	Moisture Retention	Somatosensory Evaluation					Comprehensive Score
			Appearance	Consistency	Suppleness	Skin Follows Character	Skin Retention	
①	10	10	9	8	9	9	10	65
②	15	30	9	9	7	6	8	84
③	15	20	5	4	7	7	8	66
④	15	10	2	4	8	4	8	51
⑤	15	20	9	9	7	8	8	76
⑥	10	20	2	0	7	4	8	51
⑦	10	10	5	7	7	6	8	53
⑧	0	0	0	0	0	0	0	0

**Table 4:** TEB permeability parameters under different factors.

Groups	Levels	Permeation Curves	J_ss_ (μg/cm^2^/h)	R^2^
Current density	0.1 mA/cm^2^	Q = 1.2860 ln(t) + 1.5432	1.2860	0.9636
	0.2 mA/cm^2^	Q = 2.7456 ln(t) + 2.6592	2.7456	0.9925
	0.3 mA/cm^2^	Q = 3.9801 ln(t) + 4.6427	3.9801	0.9912
	0.4 mA/cm^2^	Q = 4.6512 ln(t) + 4.8027	4.6512	0.9826
pH	4.0	Q = 3.9801 ln(t) + 4.6427	3.9801	0.9912
	5.4	Q = 3.3482 ln(t) + 2.7962	3.3482	0.9665
	6.8	Q = 1.7321 ln(t) + 2.333	1.7321	0.9969
	7.4	Q = 0.1073 ln(t) + 1.3944	0.1073	0.0939
Drug concentration	2 mg/10 cm^2^	Q = 2.7969 ln(t) + 2.9826	2.7969	0.9967
	4 mg/10 cm^2^	Q = 3.9801 ln(t) + 4.6427	3.9801	0.9912
	8 mg/10 cm^2^	Q = 4.3620 ln(t) + 5.3298	4.362l	0.9962
Drug formulations	TEB hydrogel patch with iontophoresis	Q = 3.9801 ln(t) + 4.6427	3.9801	0.9912
	TEB hydrogel patch without iontophoresis	Q = 1.4766 ln(t) + 1.6178	1.4766	0.9832
	TEB cream	Q = 0.4523 ln(t) + 0.7597	0.4523	0.9965

**Table 5:** Main pharmacokinetic parameters of TEB after transdermal administration. (n = 6).

Parameters	TEB Hydrogel Patch with Iontophoresis	TEB Hydrogel Patch without Iontophoresis	TEB Cream
AUC_(0–8 h)_ (mg/L·h)	60.99 ± 7.18 **^##^	17.71 ± 2.35	11.26 ± 1.71
MRT_(0–8 h)_ (h)	6.37 ± 0.23	5.84 ± 0.30	5.55 ± 0.36
T_max_ (h)	7.5 ± 0.89	7.00 ± 1.23	6.33 ± 2.32
C_max_ (mg/L)	18.07 ± 1.81 **^##^	4.33 ± 0.80	2.46 ± 0.39

Note: ** *p* < 0.01, as compared with the TEB hydrogel patch without iontophoresis group; ^##^ *p* < 0.01, as compared with the TEB cream group.

**Table 6:** 3-factor 2-level table.

Prescription	A: PVP K90	B: Sodium Polyacrylate/ Aluminum Glycerol	C: Gelatin
①	0.625 g	2.0 g/0.1 g	0.5 g
②	0.625 g	2.0 g/0.1 g	1.0 g
③	0.625 g	3.0 g/0.15 g	0.5 g
④	0.625 g	3.0 g/0.15 g	1.0 g
⑤	1.0 g	2.0 g/0.1 g	0.5 g
⑥	1.0 g	3.0 g/0.15 g	0.5 g
⑦	1.0 g	2.0 g/0.1 g	1.0 g
⑧	1.0 g	3.0 g/0.15 g	1.0 g

**Table 7:** Evaluation indicators of a comprehensive score for the patch.

Index Weight Score		Evaluation Criteria	Score
Initial adhesion (20 points)		Ball number ≤6	0 points
		Ball number 7–8	10 points
		Ball number 9–11	15 points
		Ball number ≥12	20 points
Moisture retention (30 points)		0~40%	0 points
		41~60%	10 points
		61~80%	20 points
		81~90%	30 points
Somatosensory evaluation	Appearance (10 points)	The color of the hydrogel patch is uniform, and the patch is evenly distributed throughout. The surface of the patch is flat, and the thickness is consistent, with no clumps or noticeable bubbles present.	10 points
		The color of the hydrogel patch is uniform, and the distribution of the patch is more even compared to before. The surface of the patch shows minimal fluctuations, but there are a few small bubbles present.	5–9 points
		The color of the hydrogel patch appears uneven, and the distribution of the patch is not uniform. There is a significant difference in thickness across the patch, and the surface of the patch may appear raised or depressed in certain areas. Additionally, there are noticeable bubbles present on the surface of the patch.	1–4 points
		Poor color, a large number of clumps, more bubbles, and so dense that the patch connot even be formed.	0 points
	Depth (10 points)	The glycerin phase and water phase are easy to mix, and the final patch can be stirred for 10 min; the final patch was difficult to stir, and then could not even be stirred. The score was divided into three grades (0~4, 4~8, and 9~10) according to the consistency.	
	Flexibility (10 points)	It is easy to coat evenly. After pressing the patch surface by hand, the patch will recover and be flat, and there will be no creases when folding the patch repeatedly. The scoring grade is divided into three levels (0~4, 4~8, and 9~10) according to the above.	
	Skin followability (10 points)	Apply the patch to the skin of the arm and wave the arm one to three times.	0–3 points
		Apply the patch to the skin of the arm and wave the arm four to nine times.	4–9 points
		Apply the patch to the skin of your arm and wave your arm 10 times.	10 points
	Skin residue (10 points)	The patch cannot be removed from the skin intact.	0 points
		The patch can be removed from the skin, leaving a large amount of residue on the surface of the skin.	1–3 points
		The patch can be completely removed from the skin, and the skin surface is slightly sticky with no visible residue, or the surface is sticky with some residue.	5–9 points
		The patch can be completely removed from the skin, and the skin surface is not sticky and residual.	10 points

## Data Availability

The data are available by contacting the corresponding author.
